# The association between lung cancer stigma and race: A descriptive correlational study

**DOI:** 10.1111/hex.13495

**Published:** 2022-04-12

**Authors:** Karen Kane McDonnell, Lisa A. Webb, Swann A. Adams, Tisha M. Felder, Rachel E. Davis

**Affiliations:** ^1^ College of Nursing University of South Carolina Columbia South Carolina USA; ^2^ College of Nursing and Arnold School of Public Health University of South Carolina Columbia South Carolina USA; ^3^ College of Nursing, Cancer Prevention & Control Program and Arnold School of Public Health University of South Carolina Columbia South Carolina USA; ^4^ Health Promotion, Education, and Behavior, Arnold School of Public Health University of South Carolina Columbia South Carolina USA; ^5^ Present address: VA North Texas HealthCare System Dallas Texas USA

**Keywords:** African American, depression, lung cancer, quality of life, stigma

## Abstract

**Background:**

Stigma is a formidable burden for survivors of lung cancer that can reduce the quality of life (QOL), resulting in physical, social and psychological challenges. This study investigates associations between stigma and depression, QOL and demographic and health‐related characteristics, including race.

**Design:**

An adapted conceptual model derived from the Cataldo Lung Cancer Stigma Scale guided this descriptive correlation study assessing stigma in African American and Caucasian survivors of lung cancer. Self‐reported, written surveys measuring depression, QOL, lung cancer stigma and demographics were administered. Statistical analysis was conducted to assess associations between stigma and depression, stigma and QOL and stigma and race, while adjusting for demographic characteristics.

**Results:**

Participants (*N* = 56) included 30 Caucasian and 26 African American survivors of lung cancer recruited from a cancer registry of an American College of Surgeons‐accredited programme, a survivors' support club and an ambulatory oncology practice in the southeastern United States. Statistical analysis yielded (1) a significant moderate positive association between depression and lung cancer stigma; (2) a significant moderate negative association between QOL and lung cancer stigma; and (3) significant relationships between race and lung cancer stigma, specifically higher degree of stigma among African Americans compared to Caucasians.

**Conclusion:**

Stigma affects many aspects of survivors' lives. Healthcare professionals need to consider how health‐related stigma may further complicate the physical burdens, psychological distresses and social challenges that accompany the disease, especially among African American survivors. Additional enquiry and interventions are needed to assist with mitigating the negative effects of stigma on survivors and their family members and friends.

**Patient or Public Contribution:**

Fifty‐six survivors of lung cancer participated in this descriptivecorrelation study. They completed written surveys measuring depression, QOL, and lung cancer stigma, plus an investigator‐developed demographic information form.

## 1 INTRODUCTION

Lung cancer is the second most prevalent cancer in men and women and the leading cause of cancer deaths in the United States.^1^, ^2^ The lung cancer incidence and mortality rate have declined due to early screening and improved treatment modalities.^1^ Mortality is directly related to stage at diagnosis because lung cancers diagnosed at early stages are more amenable to curative resection.^1^ This disease remains a major concern because of its far‐reaching negative effects on survivors' overall QOL.^3^, ^4^, ^5^, ^6^, ^7^, ^8^, ^9^, ^10^ Survivors of lung cancer, defined from the time of diagnosis to the end of life, experience significant physical symptom burden, social challenges and psychological distresses.^10^, ^11^

Survivors of lung cancer face stigma related to their diagnosis. Stigma is an ‘undesirable stereotype leading people to reduce the bearer from a whole and usual person to a tainted, discounted one.’^12^ A health‐related stigma is the perception of a behaviour that is considered unfavourable and yields an adverse result. In the case of lung cancer, the health‐related stigma is the perception that an individual with lung cancer has smoked or currently smokes cigarettes because tobacco is the leading cause of lung cancer.^4^, ^13^, ^14^, ^15^, ^16^

United States Surgeon General reports have documented strong evidence that tobacco is an individual and environmental health hazard.^17^ These reports have heightened public awareness of the links between chronic and debilitating disease processes and the behaviour of cigarette smoking. Public awareness of the hazards of smoking has led to the thought that this behaviour is an unacceptable ‘choice’ and no longer a societal norm.^18^ This leads to the ostracization of smokers and to victim‐blaming of those who formerly or currently smoke despite its known hazards.^19^, ^20^

Lung cancer stigma's layered effect on survivors can impact many aspects of their lives. Survivors who fear being stigmatized may distance themselves and refuse to disclose their illness to others, which can result in social isolation, psychological distress and delays in diagnosis and treatment.^4^, ^9^, ^19^, ^20^, ^21^ Evidence exists that stigma is an important predictor of delayed medical help‐seeking behaviours.^22^ When individuals with a potential lung cancer delay seeking help from healthcare providers, the probability of a late‐stage diagnosis increases.^22^ These effects of stigma, in turn, have a negative impact on physical health, intensifying the burden of symptoms such as shortness of breath, fatigue, insomnia and pain; the cycle continues, as physical symptoms negatively impact lung cancer survivors' ability to deal with psychological and social challenges—with all these factors together adversely affecting overall QOL and chance of survival.^4^, ^5^, ^6^, ^7^, ^13^, ^14^, ^15^, ^16^, ^17^, ^19^, ^20^, ^21^, ^22^, ^23^ In 2011, the Cataldo Lung Cancer Stigma Scale (CLCSS) became the first psychometrically tested instrument specifically designed to assess lung cancer stigma.^15^ The original psychometric testing was conducted on an online sample primarily consisting of Caucasian participants (86%); no African Americans were represented in the testing.^15^ This original evaluation yielded strong internal consistency (a Cronbach's *α* of .96), and construct validity was confirmed with correlational analysis among similar variables: depression, QOL, anxiety and social isolation.^15^ Since its development, the CLCSS has been used in more racially diverse samples. However, the African American representation in those studies has still been low, thus limiting knowledge related to lung cancer stigma among this minoritized population of lung cancer survivors.^4^, ^16^, ^17^, ^24^

Lung cancer mortality is higher among African Americans than in Caucasians.^1^ In 2021, the American Cancer Society estimated over 25,000 lung cancer diagnoses among African Americans and over 17,000 deaths resulting from this disease. The overall 5‐year relative survival rate for lung cancer is lower in African Americans in comparison to Caucasians: 16% versus 19%, respectively. Stigma (either perceived or experienced or both) negatively impacts this minoritized group of cancer survivors.^1^, ^4^, ^5^, ^6^, ^7^, ^14^, ^20^, ^24^ Given this racial disparity in lung cancer mortality, a sample with greater African American representation would add to the knowledge of lung cancer stigma and overall QOL for all lung cancer survivors. The purpose of this study was to evaluate stigma among lung cancer survivors with equal representation of Caucasians and African Americans. The aims of this study were to (1) evaluate the reliability and construct validity of the CLCSS on a purposive sample of Caucasians and African Americans with a history of lung cancer, stages I–IV; (2) calculate and compare levels of stigma, depression and QOL among demographic variables—namely, gender, race, marital status, educational attainment, income status, self‐reported health status and smoking status; and (3) evaluate the relationship between race and lung cancer stigma, adjusting for demographic variables.

The following hypotheses were tested:

1. The CLCSS will demonstrate good internal consistency among this population of lung cancer survivors, with a Cronbach's α of greater than .7.
2. There will be a statistically significant positive association between lung cancer stigma and depressive symptoms.
3. There will be a statistically significant negative association between lung cancer stigma and QOL.
4. There will be a statistically significant relationship between lung cancer stigma and African American race, adjusting for demographic variables.

## 2 MATERIALS AND METHODS

### 2.1 Design

This descriptive correlational study was guided by an adaptation of a lung cancer stigma conceptual model proposed by Cataldo et al.^14^ Cataldo and colleagues' model posits that lung cancer survivors perceive societal attitudes relative to smoking and a lung cancer diagnosis. Survivors are aware of potential or actual behaviours shown by others and by feelings that may occur because of this perception. This may lead to feelings that negatively change their identity to one of stigma and shame. The perceptions and feelings of survivors are associated with depression and lower QOL and therefore affect survival rates.

### 2.2 Participant selection, recruitment and ethical considerations

Two institutional review boards (IRBs), one with an academic affiliation and the other clinical, approved this study. Power analysis for multiple linear regression/general linear models with eight predictors was conducted in PS Power to determine a sufficient sample size for an *α* of .05, a power of 0.80 and a large effect size 0.35. This analysis indicated a desired sample size of 104 (52 African Americans and 52 Caucasians). For the Pearson correlation analysis, a sample size of 13 was required to determine whether a correlation coefficient differs from zero with an *α* value of 0.05 and a beta value of 0.2. Participants were eligible for the study if they were 21 years of age or older, had a personal history of lung cancer, self‐identified as African American or Caucasian, were residents of South Carolina and could speak and read English. The primary recruitment method was an IRB‐approved recruitment invitation describing the purpose and procedure of the study. The invitations were mailed over a 4‐month period to 500 potential participants identified by a cancer registry from a local cancer centre accredited by the American College of Surgeons. A follow‐up telephone call was made to potential participants within 1–2 weeks of the mailing. A secondary recruitment method involved a local support club for survivors of lung cancer. This avenue provided the opportunity to meet potential participants face to face after a club meeting. A third and final recruitment effort occurred at a large, private ambulatory oncology practice. IRB‐approved flyers were placed in strategic areas at this practice, and a research team member was introduced during scheduled appointments. Written informed consent was obtained from all participants. Upon study completion, each participant received an appreciation gift (a gift card to a local retail store).

## 3 INSTRUMENTS

A demographic form was used to collect information, including gender, age, race, annual household income, year of cancer diagnosis, self‐rated health status, smoking status, educational attainment and employment status. Race was self‐reported and was examined because lung cancer mortality rates are currently the highest among African Americans in the United States. These data allowed for evaluation of race—a social construct serving as a proxy measure of exposure to racism and related structural inequities—and the ways in which it may influence lung cancer survivors' experiences and outcomes.^25^ In addition, three instruments were used to measure variables of interest.

### 3.1 Stigma

The CLCSS is a 31‐item, 4‐point Likert scale that evaluates stigma.^15^ The original psychometric testing was conducted by Cataldo et al.^14^, ^15^ Exploratory factor analysis identified four domains: stigma and shame, social isolation, discrimination and smoking. Reliability was established by a coefficient *α* of .96 for the entire scale. Construct validity was supported by association with the related constructs of self‐esteem, depression, social support and social conflict. CLCSS scores range from 31 (low stigma) to 124 (high stigma).

### 3.2 Depression

The Center for Epidemiologic Studies Depression Scale (CES‐D) is a 20‐item, nondiagnostic, self‐report scale.^26^ The instrument assesses several domains of depression, including depressed mood, feelings of guilt and worthlessness, feelings of helplessness and hopelessness, psychomotor hindrances, loss of appetite and sleep disturbance.^26^ This instrument has been shown to be reliable across gender, race and age, with a high internal consistency, ranging from 0.85 to 0.90.^26^ Scores for this instrument range from 0 (indicating low depressive symptoms) to 60 (high depressive symptoms).

### 3.3 Quality of life

The European Organization for Research and Treatment of Cancer Quality of Life Questionnaire Core 30 (EORTC QLQ‐C30) was tested on patients with lung cancer and yielded an acceptable internal consistency of 0.70 with a sample size of 110.^27^ Criterion validity was supported by correlation with clinical parameters that addressed all domains of the instrument.^27^ A 13‐item scale specifically for lung cancer was also used. The score range for this instrument is 0 (low global health) to 100 (high global health); 56.6 is the mean for lung cancer survivors.

### 3.4 Data analysis

Data were analysed using SPSS Statistics Version 25.0®. Descriptive statistics (means and frequencies) were calculated to describe the sample. Means were compared using *t* tests or one‐way analysis of variance as appropriate. The CLCSS was reverse‐scored and averaged. The CES‐D was scored and averaged according to the Center for Epidemiologic Studies' guidelines. Participants' QOL was calculated by applying linear transformation to two global health questions. Construct validity of the CLCSS was evaluated using correlational analysis, which examined the linear relationship between lung cancer stigma and depressive symptoms, and between lung cancer stigma and self‐rated health. General linear models were used to estimate the association between lung cancer stigma and race, with other demographic characteristics added as covariates in separate models testing each of these additional characteristics individually. Factor and covariate model effects were applied using the general linear model option in SPSS. Due to the reduced sample size and subsequent reduction in power, multiple bivariable models were run initially, which included race plus another single demographic variable, using lung cancer stigma as the dependent variable. Using an *α* cut point of .05 from the bivariable models, we determined the covariates that may significantly confound the relationship between race and stigma. The final model included race and any other independent variable that demonstrated a significant relationship. Significance in the final linear model was determined at *p* < .05.

## 4 RESULTS

### 4.1 Participant profile

A total of 62 individuals participated. Fifty of those 62 participants (80.6%) were recruited from the 500‐member cancer registry direct mailing. Of the 500 direct mailings, 48 were returned and 178 recipients could not be contacted. Overall, this response reflects an 18.2% recruitment rate through direct mail. The remaining 12 participants were recruited by the two other recruitment strategies. Six participants did not complete all the requested survey material and therefore were excluded from the analysis. Of the 56 participants with complete data, 30 were Caucasians (54%) and 26 were African Americans (46%). Participant ages ranged from 48 to 81 years, with a mean of 67 (SD = 8.9). Over half of the sample was married and had some college education. Participants' lung cancer stages ranged from IA to IV, with over half of the participants diagnosed within the past 2–3 years. Over half of the participants rated their health as fair to poor. The sample included 43 former smokers, seven never smokers and six current smokers (see Table 1).

**Table 1:** Demographic characteristics

Variable	Possible value	Self‐reported results
Age (years), mean (SD; range)		68.11 (9.451; 45–89)
Gender, *n* (%)	Male	19 (34)
	Female	37 (66)
Race, *n* (%)	Caucasian	30 (54)
	African American	26 (46)
Marital status, *n* (%)	Single	5 (9)
	Married	31 (55)
	Separated/divorced	11 (20)
	Widowed	9 (16)
Education, *n* (%)	Some high school	8 (14)
	High school graduate or General Educational Development	10 (18)
	Some college (13 years)	22 (39)
	College (≥4 years)	16 (29)
Employment status, *n* (%)	Employed for wages	6 (11)
	Self‐employed	2 (4)
	Out of work < 1 years	2 (4)
	Out of work > 1 years	3 (5)
	Homemaker	1 (2)
	Retired	27 (48)
	Unable to work	15 (27)
Annual household income ($), *n* (%)	<5000	1 (2)
	5000–9999	6 (11)
	10,000–19,999	8 (14)
	20,000–49,999	23 (41)
	50,000–100,000	9 (16)
	>100,000	6 (11)
	Not reported	3 (5)
Health status, *n* (%)	Excellent	2 (4)
	Very good	6 (11)
	Good	18 (32)
	Fair	21 (37)
	Poor	9 (16)
Year of lung cancer diagnosis, *n* (%)	2016 or later	30 (48)
	2015	13 (21)
	2014	2 (3)
	2013	2 (3)
	2012	1 (2)
	2011	7 (11)
	2010 or before	7 (11)
Smoking status, *n* (%)	Never smoker	7 (12)
	Former smoker	43 (77)
	Current smoker	6 (11)

*Note*: Some categories may not sum to 100% due to rounding.

### 4.2 Analysis

The CLCSS had a Cronbach's *α* of .96, indicating very good internal consistency. Correlational analysis yielded a statistically significant moderate positive association between stigma and depression (*r* = .345, *p* = .005) and a statistically significant moderate negative association between stigma and QOL (*r* = −.303, *p* = .012). These findings support hypotheses 1–3 (see Table 2). The average stigma score for Caucasian participants was lower than the average stigma score for African Americans (as shown in Table 2).

**Table 2:** Correlation between stigma, depression and QOL

Measure (Instrument)	Lung cancer stigma (CLCSS)	Depressive symptoms (CES‐D)	Global health/QOL (EORTC QLQ‐C30)
Lung cancer stigma (CLCSS)		.345*	−.303**
Depressive symptoms (CES‐D)	.345*		−.523*
Global health/QOL (EORTC QLQ‐C30)	−.303**	−.523*	

Abbreviations: CES‐D, Center for Epidemiologic Studies Depression Scale; CLCSS, Cataldo Lung Cancer Stigma Scale; EORTC QLQ‐C30, European Organization for Research and Treatment of Cancer Quality of Life Questionnaire Core 30; QOL, quality of life.

* *p* = .05.

** *p* = .01.

The independent *t* test showed a *t* value of −3.3 (*p* = .002) and an *η* ^2^ of 16%, indicating that race had a huge effect on the mean stigma scores. Race and gender were found to be insignificantly related to depression and QOL (see Table 3).

**Table 3:** Differences in stigma, depression and QOL for demographic and health‐related characteristics

Demographic variable	Value	Instrument mean scores (SD)^a^		
		Stigma (CLCSS)	Depression (CES‐D)	Global health/QOL (EORTC QLQ‐C30)
Overall		51.38 (16.32)	16.3 (11.8)	62.68 (24.03)
Race	Caucasian	45.2 (14.96)*	15.43 (13.55)	61.67 (22.89)
	African American	58.5 (15.10)*	17.31 (9.57)	63.85 (25.71)
Gender	Male	51.36 (17.30)	13.53 (8.67)	59.53 (28.55)
	Female	51.37 (16.04)	17.73 (13)	64.30 (21.62)
Marital status	Single	68.2 (14.97)*	12.8 (12.55)	84.8 (17.22)*
	Married	47.45 (14.77)	16.71 (13.13)	62.90 (21.69)
	Separated/divorced	58.28 (14.92)	20.09 (7.18)*	50.45 (23.69)
Health status	Excellent	50 (26.87)	13 (9.9)	58 (11.31)
	Very good	46.17 (13.15)	9.33 (8.57)	88.5 (8.52)
	Good	45.5 (17.54)	15.72 (15.96)	68.6 (14.72)
	Fair	56.24 (15.02)	18.81 (8.59)	58.09 (24.78)
	Poor	55.56 (15.33)	17 (10.81)	45.33 (30.61)
Smoking status	Current	52.67 (22.21)	17.17 (9.95)	59.5 (27.91)
	Former	52.47 (16.04)	16.84 (12.31)	61.35 (24.49)
	Never	43.57 (12.2)	12.28 (10.61)	73.57 (16.10)

Abbreviations: CLCSS, Cataldo Lung Cancer Stigma Scale; CES‐D, Center for Epidemiologic Studies Depression Scale; EORTC QLQ‐C30, European Organization for Research and Treatment of Cancer Quality of Life Questionnaire Core 30; QOL, quality of life.

^a^ Scale ranges: stigma, 31–124; depression, 0–60; QOL, 0–100.

* *p* < .05.

Participants who were married showed lower lung cancer stigma scores on average (47.45 ± 14.77) compared to their single (68.2 ± 14.97) and separated or divorced (58.28 ± 14.92) counterparts. This finding was statistically significant, with a large effect related to marital status (*p* = .033, *η* ^2^ = 0.17). Marital status was also found to have a statistically significant relationship with QOL. On average, participants who were single showed a higher QOL (84.8 ± 17.22) than participants who were separated or divorced (50.45 ± 23.69). This was shown to be significant statistically, with a large effect attributed to marital status (*p* = .039, *η* ^2^ = 0.17). A generalized linear model was used to evaluate relationships among demographic variables and lung cancer stigma. The final model consisted of race and income. Race was found to have a statistically significant relationship with lung cancer stigma (Wald *χ* ^2^ = 9.62, *p* = .002), which partially supports hypothesis 4. Income was not statistically associated with lung cancer stigma (Wald *χ* ^2^ = 6.27, *p* = .12). The estimated marginal mean for lung cancer stigma for Caucasians was 48.05; for African Americans, it was 60.03 (see Table 4).

**Table 4:** Lung cancer stigma: adjusted mean for race

Independent variable	Adjusted mean		*p* Value
	Caucasians (*n* = 30)	African Americans (*n* = 26)	
Race	48.05	60.03	.002

*Note*: Adjusted for gender, marital status, educational attainment, work status, self‐reported health, time of diagnosis and smoking status.

## 5 DISCUSSION

### 5.1 Evaluation of the CLCSS

In this study, the CLCSS was found to be a reliable and valid instrument among the sample of lung cancer survivors. The instrument's strong Cronbach's *α*, though indicating good internal consistency, may also signal redundancy in items. Carter‐Harris and Hall^19^ developed a shortened version of the CLCSS. This shorter version consisted of 21 items and indicated high internal consistency, with a Cronbach's *α* of .93. Although the shortened version demonstrated comparable reliability and validity, the CLCSS was the instrument of choice for this study. It is the first psychometrically validated lung cancer stigma instrument, allowing this study to provide foundational knowledge relative to lung cancer stigma and African American survivors of lung cancer. Further investigation with an even shorter instrument is warranted and would continue to add to existing knowledge of lung cancer stigma. It is possible that a shortened version would be more widely accepted in clinical practice, as it may lessen the time and burden on survivors and providers.

### 5.2 Relationship of stigma with depression and QOL

Lung cancer survivors often experience psychological distress as a result of their disease. This study presents statistically significant findings of a moderate positive association between stigma and depression. Depressive symptoms are prevalent among those living with lung cancer, and depression is considered one of the most prominent psychological challenges for this population.^5^, ^7^, ^13^, ^14^ In their study (*N* = 190), Cataldo et al.^14^ found that increased depression was strongly associated with increased stigma and that depression was secondary to lung cancer stigma in impact on QOL. The two variables together explain most of the variance in QOL. It is imperative to consider the interaction of depression and stigma, and how it may negatively impact social challenges and physical symptoms, thereby negatively affecting overall QOL. This is especially important among African American cancer survivors because depression is frequently underdiagnosed, misdiagnosed and undertreated.^20^ Untreated depression is likely to have a further negative impact on all areas of life, potentially leading to an inability to manage physical symptom burden and impeding the adoption of health behaviours that can help improve overall QOL.^21^

QOL is multifaceted and can have a direct influence on survival rates of cancer survivors.^22^ This study demonstrated that lung cancer stigma has a significantly moderate negative association with QOL. Chambers et al.^5^ found that lung cancer stigma had an adverse effect on overall QOL in their systematic review. Cataldo et al.^14^ obtained the same finding and indicated that stigma was a major contributor to the variance in QOL. Given that survival rates are increasing among lung cancer survivors, it is essential to consider the effect of lung cancer stigma, including the effect that it has on overall QOL, along with the daily challenges faced by these individuals.

### 5.3 Relationship of stigma with demographic characteristics

In this study, African Americans reported higher lung cancer stigma and depression scores compared to Caucasians and race significantly accounted for the variance in lung cancer stigma scores. Comparatively, the average overall QOL among African Americans was higher than for Caucasians in this study. This may be due, at least partly, to how QOL is calculated with this instrument.^15^ Two weighted questions situated towards the end of the instrument focus on overall health and overall QOL within the past week. This can create a wide variation in the interpretation of the questions and thus in responses. African American participants in this study assigned a higher score for QOL despite their high physical symptom burden in comparison to Caucasian participants. Rao et al.^24^ documented similar findings among patients with breast, colon, head/neck and lung cancers. Further enquiry from an asset model approach that centres on the positive QOL perspectives of African Americans could generate a better understanding of this finding.

Demographic characteristics showed some important associations with the three dependent variables of stigma, depression and QOL. As previously noted, race was significantly associated with stigma and depression. However, other characteristics—such as gender, educational attainment, employment status and self‐rated health status—did not have a significant relationship with stigma, depression or QOL. Although there was no significant finding on smoking status and its relationship with stigma, depression or QOL, the never smokers in the study did, on average, show a lower lung cancer stigma score in comparison to current and former smokers. This finding is similar to those reported by Cataldo et al.^14^ Marital status showed a significant relationship with stigma, depression and QOL in the descriptive analysis. Participants who were single reported a higher level of stigma and QOL and a low level of depressive symptoms, whereas divorced or separated participants reported a higher level of depression. Interestingly, marital status was not related to stigma in the general linear model. The Caucasians in this sample had an average stigma score that was 10.8 points lower than African Americans, indicating a lower experience of stigma on average. Individuals in this sample whose household income was above $20,000 annually had a lower stigma score of 11.1 (on average) in comparison to individuals earning under $20,000 annually, which is considered poverty level for a household of three people.^28^ Socioeconomic status, race, education and geographic location are factors often associated with higher rates of mortality.^29^, ^30^

For survivors, race showed a relationship with lung cancer stigma. Income did not. This is interesting due to the geographical location in which the sample was obtained—the southeastern United States, known for seven out of the top 10 states with the highest poverty rates (Retrieved from https://www.forbes.com/sites/andrewdepietro/2021/11/04/us-poverty-rate-by-state-in-2021/?sh=29e145111b38). Low socioeconomic status and African American race have been known drivers for negative impact with individuals with cancer.^31^ This is true even more so with African Americans with lung cancer. Webb and McDonnell^32^ found that African Americans living with lung cancer felt that the diagnosis was considered a death sentence, in part by the perceived stigma that these survivors experienced. These relationships may be due in part to the higher rates of smoking in this area of the country, which may be linked to relaxed smoking laws and policies.^33^ Tobacco use and exposure to secondhand smoke remain leading public health threats in states in the Southeast, also known as America's tobacco belt.^33^ African Americans historically have experienced negative associations with racial discrimination, which substantially correlates with being stigmatized.^29^, ^30^, ^31^ The perception of stigma and discrimination can lead to depression, anxiety, social isolation, delayed medical care and altered patient–provider communication.^7^, ^13^, ^14^, ^34^, ^35^, ^36^, ^37^, ^38^ This indicates the vital need for future research to assess the roles that structural and interpersonal racism and discrimination play in relation to stigma, and the psychological, physical and social challenges associated with living with a lung cancer diagnosis.

Other researchers agree that conducting research with survivors of lung cancer is challenging, primarily because of low recruitment rates resulting from patient‐, physician‐, protocol‐ and healthcare system‐related barriers.^39^ In this study, recruitment through the cancer registry was the most successful strategy. The recruitment rate was comparable to our other studies recruiting survivors of localized lung cancer and their family members (14%–20%).^32^, ^36^, ^37^, ^40^ This study recruited survivors of all stages who described their health as fair to poor (*n* = 30, 54%), which may have hindered recruitment rates. Other barriers may have included a short study time frame (4–6 months) and the research topic of stigma. The topic of stigma alone may promote feelings of anxiety and depression, which limits the ease of conversation about it. The discussion of health‐related stigma may result in an unexpected negative impact on the survivor. This study indicates that African Americans with lung cancer experience higher levels of stigma. Additional investigation of lung cancer stigma in this population will assist with the development of multilevel interventions to alleviate this burden.

## 6 CONCLUSION

There are limitations to this study. The sample size of 56 was small and yielded a low power for the regressional/general linear model. While this prevented us from drawing any conclusions from null findings, it does not invalidate any significant findings. Indeed, it suggests that the association is quite strong if it reaches statistical significance in an underpowered study. In addition, participants were drawn from a nonrepresentative convenience sample recruited from a cancer registry, a support club and an ambulatory outpatient clinical practice near one another within one southeastern state, thereby limiting generalizability. Lung cancer survivors in this region may differ from survivors in other areas of the state, country or globe. Finally, this study did not evaluate the potential role of racism and discrimination. Future studies on lung cancer stigma should include measures of racism and focus on inclusive recruitment approaches to ensure representative research participation of African Americans and other minoritized populations worldwide.

Stigma affects many aspects of the lives of survivors of lung cancer. It is imperative that healthcare professionals, when providing care and recommendations to these survivors, take into consideration how stigma may negatively impact the experiences of their patients, particularly African American lung cancer survivors. The effect that stigma has on lung cancer survivorship may further complicate physical burdens, psychological distresses and social challenges.

To our knowledge, our study is the first to evaluate lung cancer stigma, with nearly 50% representation of African Americans among a population of lung cancer survivors, compared to previous studies ranging from 14% to 17%.^14^, ^15^, ^19^ It is vital to increase the participation of African Americans and other minoritized populations in cancer survivorship research to generate the highest standards of scientific evidence and achieve cancer equity for all.^41^ These findings will add to the knowledge of lung cancer stigma for all survivors of lung cancer, and particularly, African Americans.

## AUTHOR CONTRIBUTIONS

Karen K. McDonnell, Lisa A. Webb, Swann A. Adams, Tisha M. Felder and Rachel E. Davis collaborated on the design of the study and funding support. Lisa A. Webb and Karen K. McDonnell draughted the manuscript. Karen K. McDonnell prepared the manuscript for submission. All authors reviewed and approved the final manuscript.

## CONFLICTS OF INTEREST

The authors declare no conflicts of interest.

## Data Availability

The data that support the findings of this study are available from the corresponding author upon reasonable request.

## References

[1] American Cancer Society. Cancer facts and figures. 2021. Accessed May 16, 2021. https://www.cancer.org/content/dam/cancer-org/research/cancer-facts-and-statistics/annual-cancer-facts-and-figures/2021/cancer-facts-and-figures-2021.pdf

[2] Bade BC , Cruz CSD . Lung cancer 2020: epidemiology, etiology, and prevention. Clin Chest Med. 2020;41(1):1‐24. 32008623 10.1016/j.ccm.2019.10.001

[3] Brown‐Johnson CG , Cataldo JK , Orozco N , Lisha NE , Hickman NJ , Prochaska JJ . Validity and reliability of the internalized stigma of smoking inventory: an exploration of shame, isolation, and discrimination in smokers with mental health diagnoses. Am J Addict. 2015;24(5):410‐418. 10.1111/ajad.12215 25930661 PMC4675843

[4] Chambers SK , Baade P , Youl P , et al. Psychological distress and quality of life in lung cancer: the role of health‐related stigma, illness appraisals and social constraints. Psychooncology. 2015;24(11):1569‐1577. 10.1002/pon.3829 25920906 PMC5029590

[5] Chambers SK , Dunn J , Occhipinti S , et al. A systematic review of the impact of stigma and nihilism on lung cancer outcomes. BMC Cancer. 2012;12(1):184. 10.1186/1471-2407-12-184 22607085 PMC3517321

[6] Chapple A , Ziebland S , McPherson A . Stigma, shame, and blame experienced by patients with lung cancer: qualitative study. BMJ. 2004;328(7454):1470. 10.1136/bmj.38111.639734.7C 15194599 PMC428516

[7] Gonzalez BD , Jacobsen PB . Depression in lung cancer patients: the role of perceived stigma. Psychooncology. 2012;21(3):239‐246. 10.1002/pon.1882 22383265

[8] Gonzalez BD , Jim HSL , Cessna JM , Small BJ , Sutton SK , Jacobsen PB . Concealment of lung cancer diagnosis: prevalence and correlates. Psychooncology. 2015;24(12):1774‐1783. 10.1002/pon.3793 25753772 PMC4564357

[9] Hamann HA , Ostroff JS , Marks EG , Gerber DE , Schiller JH , Lee SJC . Stigma among patients with lung cancer: a patient‐reported measurement model. Psychooncology. 2014;23(1):81‐92. 10.1002/pon.3371 24123664 PMC3936675

[10] Houlihan NG , Tyson LB . Lung Cancer. 2nd ed. Oncology Nursing Society; 2012.

[11] Institute of Medicine and National Research Council . From Cancer Patient to Cancer Survivor: Lost in Transition. National Academies Press; 2006. 10.17226/11468

[12] Goffman E . Stigma: Notes on the Management of Spoiled Identity. Touchstone Publishing; 1963.

[13] Brown‐Johnson CG , Brodsky JL , Cataldo JK . Lung cancer stigma, anxiety, depression, and quality of life. J Psychosoc Oncol. 2014;32(1):59‐73. 10.1080/07347332.2013.855963 24428251 PMC4634635

[14] Cataldo JK , Jahan TM , Pongquan VL . Lung cancer stigma, depression, and quality of life among ever and never smokers. Eur J Oncol Nurs. 2012;16(3):264‐269. 10.1016/j.ejon.2011.06.008 21803653 PMC3360805

[15] Cataldo JK , Slaughter R , Jahan TM , Pongquan VL , Hwang WJ . Measuring stigma in people with lung cancer: psychometric testing of the Cataldo Lung Cancer Stigma Scale. Oncol Nurs Forum. 2011;38(1):E46‐E54. 21186151 10.1188/11.ONF.E46-E54 PMC3182474

[16] Carter‐Harris L , Hall LA . Development of a short version of the Cataldo Lung Cancer Stigma Scale. J Psychosoc Oncol. 2014;32(6):665‐677. 10.1080/07347332.2014.955238 25157591 PMC4224672

[17] U.S. Department of Health and Human Services . The health consequences of smoking—50 years of progress: a report of the Surgeon General Office on Smoking and Health. Department of Health and Human Services, Centers for Disease Control and Prevention, National Center for Chronic Disease Prevention and Health Promotion. 2014. Accessed May 16, 2021. http://www.surgeongeneral.gov/library/reports/50-years-of-progress/exec-summary.pdf

[18] Bayer R . Stigma and the ethics of public health: not can we but should we. Soc Sci Med. 2008;67(3):463‐472. 10.1016/j.socscimed.2008.03.017 18502551

[19] Shen MJ , Coups EJ , Li Y , Holland JC , Hamann HA , Ostroff JS . The role of posttraumatic growth and timing of quitting smoking as moderators of the relationship between stigma and psychological distress among lung cancer survivors who are former smokers. Psychooncology. 2015;24(6):683‐690. 10.1002/pon.3711 25345591 PMC4515573

[20] Rauma V , Sintonen H , Räsänen JV , Salo JA , Ilonen IK . Long‐term lung cancer survivors have permanently decreased quality of life after surgery. Clin Lung Cancer. 2015;16(1):40‐45. 10.1016/j.cllc.2014.08.004 25450878

[21] Rowland C , Danson SJ , Rowe R , et al. Quality of life, support and smoking in advanced lung cancer patients: a qualitative study. BMJ Support Palliat Care. 2016;6(1):35‐42. 10.1136/bmjspcare-2013-000589 24785651

[22] Carter‐Harris L , Hermann CP , Schreiber J , Weaver MT , Rawl SM . Lung cancer stigma predicts timing of medical help‐seeking behavior. Oncol Nurs Forum. 2014;41(3):E203‐E210. 10.1188/14.ONF.E203-E210 24769603 PMC4160058

[23] Montazeri A . Quality of life data as prognostic indicators of survival in cancer patients: an overview of the literature from 1982 to 2008. Health Qual Life Outcomes. 2009;7(102):1‐21. 10.1186/1477-7525-7-102 20030832 PMC2805623

[24] Rao D , Debb S , Blitz D , Choi SW , Cella D . Racial/ethnic differences in the health‐related quality of life of cancer patients. J Pain Symptom Manage. 2008;36(5):488‐496. 10.1016/j.jpainsymman.2007.11.012 18504096 PMC2596636

[25] Boyd RL , Lindo EG , Weeks LD , McLemore MR . On racism: a new standard for publishing on racial health inequities. Health Affairs Blog . 2020. 10.1377/hblog20200630.939347

[26] Radloff LS . The CES‐D Scale: a self‐report depression scale for research in the general population. Appl Psychol Meas. 1977;1(3):385‐401. 10.1177/014662167700100306

[27] Nicklasson M , Bergman B . Validity, reliability, and clinical relevance of EORTC QLQ‐C30 and LC13 in patients with chest malignancies in a palliative setting. Qual Life Res. 2007;16(6):1019‐1028. 10.1007/s11136-007-9210-8 17479356

[28] U.S. Department of Health and Human Services . 2019 poverty guidelines. Office of the Assistant Secretary for Planning and Evaluation. 2019. Accessed May 16, 2021. https://aspe.hhs.gov/2019-poverty-guidelines

[29] Dixit N , Crawford GB , Lemonde M , Rittenberg CN , Fernández‐Ortega P . Left behind: cancer disparities in the developed world. Support Care Cancer. 2016;24(8):3261‐3264. 10.1007/s00520-016-3192-4 27052305

[30] Singh GK , Jemal A . Socioeconomic and racial/ethnic disparities in cancer mortality, incidence, and survival in the United States, 1950‐2014: over six decades of changing patterns and widening inequalities. J Environ Public Health . 2017. 10.1155/2017/2819372 PMC5376950 28408935

[31] Green PM , Guerrier‐Adams S , Okunji PO , Schiavone D , Smith JE . African American health disparities in lung cancer. Clin J Oncol Nurs. 2013;17(2):180‐186. 10.1188/13.CJON.180-186 23538254

[32] Webb LA , McDonnell KK . Not a death sentence: perspectives of African American women living with lung cancer. Oncol Nurs Forum. 2018;45(1):46‐54. 10.1188/18.ONF.46-54 29251297

[33] American Lung Association . State of tobacco control. 2021. Accessed May 16, 2021. https://www.lung.org/research/sotc/state-grades/south-carolina

[34] Traeger L , Cannon S , Pirl WF , Park ER . Depression and undertreatment of depression: potential risks and outcomes in black patients with lung cancer. J Psychosoc Oncol. 2013;31(2):123‐135. 10.1080/07347332.2012.761320 23514250 PMC4360992

[35] Chambers SK , Morris BA , Clutton S , et al. Psychological wellness and health‐related stigma: a pilot study of an acceptance‐focused cognitive behavioural intervention for people with lung cancer. Eur J Cancer Care. 2015;24(1):60‐70. 10.1111/ecc.12221 PMC4309461 25053458

[36] McDonnell KK , Owens OL , Messias DKH , et al. After ringing the bell: receptivity of and preferences for healthy behaviors in African American dyads surviving lung cancer. Oncol Nurs Forum. 2020;47(3):281‐291. 10.1188/20.ONF.281-291 32301934

[37] McDonnell KK , Owens OL , Hilfinger Messias DK , et al. Health behavior changes in African American family members facing lung cancer: tensions and compromises. Eur J Oncol Nurs. 2019;38:57‐64. 10.1016/j.ejon.2018.12.002 30717937

[38] Shen MJ , Hamann HA , Thomas AJ , Ostroff JS . Association between patient‐provider communication and lung cancer stigma. Support Care Cancer. 2016;24(5):2093‐2099. 10.1007/s00520-015-3014-0 26553030 PMC4805469

[39] Curran WJ Jr. , Schiller JH , Wolkin AC , Comis RL , Scientific Leadership Council in Lung Cancer of the Coalition of Cancer Cooperative Groups . Addressing the current challenges of non‐small‐cell lung cancer clinical trial accrual. Clin Lung Cancer. 2008;9(4):222‐226. 18650170 10.3816/CLC.2008.n.033

[40] McDonnell KK , Gallerani DG , Newsome BR , et al. A prospective pilot study evaluating feasibility and preliminary effects of breathe easier: a mindfulness‐based intervention for survivors of lung cancer and their family members (dyads). Integr Cancer Ther. 2020;19:1‐14. 10.1177/1534735420969829 PMC7604980 33118443

[41] Lett E , Asabor E , Beltrán S , Michelle Cannon A , Arah OA . Conceptualizing, contextualizing, and operationalizing race in quantitative health sciences research. Ann Fam Med. Published online January 19, 2202;20(2):157‐163. 10.1370/afm.2792 PMC8959750 35045967

